# Chronic Exposure to the Combination of Cigarette Smoke and Morphine Decreases CD4^+^ Regulatory T Cell Numbers by Reprogramming the Treg Cell Transcriptome

**DOI:** 10.3389/fimmu.2022.887681

**Published:** 2022-04-20

**Authors:** Ying Shao, William Cornwell, Keman Xu, Aaron Kirchhoff, Fatma Saasoud, Yifan Lu, Xiaohua Jiang, Gerard J. Criner, Hong Wang, Thomas J. Rogers, Xiaofeng Yang

**Affiliations:** ^1^ Cardiovascular Research Center, Lewis Katz School of Medicine at Temple University, Philadelphia, PA, United States; ^2^ Center for Inflammation and Lung Research, Lewis Katz School of Medicine at Temple University, Philadelphia, PA, United States; ^3^ Center for Metabolic Disease Research, Lewis Katz School of Medicine at Temple University, Philadelphia, PA, United States; ^4^ Department of Thoracic Medicine and Surgery, Lewis Katz School of Medicine at Temple University, Philadelphia, PA, United States

**Keywords:** CD4^+^CD25^high^CD127^low^ regulatory T cells (Treg), cigarette smoke, morphine, smoke plus morphine, RNA-Seq transcriptomes

## Abstract

There is a high incidence of tobacco use among intravenous opioid drug users. It is well established that opioids and tobacco smoke induce a degree of immune activation, and recent work suggests that the combination of these drugs promotes further activation of the immune system. Our approach involved the treatment of wild-type mice with cigarette smoke (SM) for a period of eight weeks, and the chronic continuous administration of morphine (M) *via* mini-pumps for the final four weeks. In an effort to examine the responses of CD4^+^CD25^high^CD127^low^ regulatory T (Treg) cells, the major immune suppressive cell type, to the combined chronic administration of SM and M, we determined the frequency of these cells in the spleen, lymph nodes and lungs. Flow cytometric analyses showed that SM and M individually, and the combination (SM + M) have differential effects on the numbers of Treg in the spleen, lymph node, and lung. Either SM or M alone increased Treg cell numbers in the spleen, but SM+M did not. Furthermore, SM + M decreased Treg cell numbers in the lymph node and lung. We then performed RNA-Seq on Treg cells from mice treated with SM, M, or SM + M, and we found that the S + M induced a number of significant changes in the transcriptome, that were not as apparent following treatment with either SM or M alone. This included an activation of TWEAK, PI3K/AKT and OXPHOS pathways and a shift to Th17 immunity. Our results have provided novel insights on tissue Treg cell changes, which we suggest are the result of transcriptomic reprogramming induced by SM, M, and SM + M, respectively. We believe these results may lead to the identification of novel therapeutic targets for suppressing smoke and opioid induced Treg cell impairment.

## Introduction

Tobacco use is a major cause of death from cancer, cardiovascular disease, and pulmonary disease (1) mediated in part *via* 11 oncogenic pathways (2), signals from 29 particulate elements (3), a variety of DNA adducts (4, 5), and more than 5,000 chemicals (6, 7). In chronic obstructive pulmonary disease (COPD) patients, higher numbers of Treg cells have been found in the pulmonary lymphocyte follicles but not in the lung parenchyma (8). Furthermore, Foxp3^+^ Treg are upregulated in large airways but downregulated in small airways in patients with COPD which correlates with limited airflow (9, 10). SM extracts promote DNA methylatransferase 3a expression in dendritic cells, resulting in increased Th17 cell numbers and fewer Treg cells (11). In the early development of COPD in animal models, exposure to SM decreases the numbers of signal transducer and activator of transcription 5 (STAT5) and phospho-STAT5 (pSTAT5) expressing cells and the expression levels of Treg cytokines transforming growth factor- β (TGF-β) and interleukin-10 (IL-10). However SM exposure later increases the numbers of STAT3^+^ and pSTAT3^+^ cells and Th17 cytokine IL-17 (12). We previously reported that the number of Treg cells in the blood of patients with severe COPD was significantly reduced (13), suggesting that chronic inflammation may be promoted by the lack of Treg cells. However, the molecular basis for the change in Treg numbers at the level of the transcriptome remains unknown.

In addition, people who use illicit opiate drugs are reported to have high rates of tobacco use. In a 24-year follow-up of narcotics addicts, the death rate of smokers were four times than of non‐smokers (14, 15). Morphine, a breakdown product from heroin, has been found to impair innate immune responses, T cell activation, and shift toward type 2 T helper cell (Th2) differentiation with increased Th1 cell death (16). We have reported that opioids upregulate the expression of several important pro-inflammatory chemokines as well as the chemokine receptor C-C Motif Chemokine Receptor 5 (CCR5) on T cells, monocytes and macrophages (17). In addition, morphine can increase IL-10 levels (18) and concomitantly reduce IL-17 secretion (19) from cultured CD4^+^ T cells (20). However, molecular mechanisms underlying morphine effects on Treg homeostasis and function remain poorly defined.

In this study, we report the Treg changes in lung, lymph node and spleen, and accompanying transcriptomic reprogramming of Treg cells upon the administration of SM, M, and the combination of SM with M. The combination of SM plus M decreases Treg cells in lymph node and lung potentially by modulating cytokine expression, and cell death pathways. Since Treg cells are the major immunosuppressive cell type and play essential roles in maintaining immune homeostasis, and inhibiting inflammation, autoimmune diseases and anti-tumor immune response, novel therapeutic strategies could be developed to maintain Treg survival (21, 22).

## Materials and Methods

### Animals

B6.129S4-*Arg1^tm1.1Lky^*/J mice were purchased from the Jackson Laboratory (Stock No: 015857, Bar Harbor, ME). All mice were weaned at 3 weeks of age and maintained on chow diet until 8 weeks old. All animal experiments were performed in accordance with the Institutional Animal Care and Use Committee (IACUC) Guidelines and Authorization for the use of Laboratory Animals and were approved by the IACUC of Temple University Lewis Katz School of Medicine.

### Cigarette Smoke Administration

SM from filtered 3R4F Cigarettes (University of Kentucky, Lexington, KY) was delivered using a TED-10 smoking system (Teaque Enterprises, Woodland, CA) using standard parameters (23). Mice were exposed in whole body chambers to mainstream SM as previously described (15). Mice were exposed to SM for two hours, 5 days a week for a total of 8 weeks. Control mice, not exposed to cigarette smoke, were exposed to filtered room air in identical whole body chambers for the same time period. An average total particulate matter (TPM) for the first week was 150 mg/day, and this was elevated to 225 mg/day beginning with the second week and for the remainder of the experiment. This dosing regimen is sufficient to establish an inflammatory response in the lung tissue.

### Morphine Administration *via* Mini−Pumps

Morphine administration was conducted using 28 day Alzet mini-pumps (Durect Corp., Cupertino, CA). These pumps deliver a consistent and continuous dose of morphine over a 28-day-period. The animals received a dose of 1 mg/kg/day.

### Cell Preparation and Flow Cytometry

To make single cell suspension from spleen and lymph node (LN), freshly collected spleen and LN were washed with PBS and homogenized gently between the frosted ends of the slides in FACS buffer. The suspended cells were transferred into a 15 ml conical tube and immediately centrifugated at 600 g for 5 minutes at 4°C, then the supernatant was removed. The red blood cells (RBC) in splenic cell pellets were lyzed by adding 1 ml ACK lysis buffer and incubated for 5 minutes, then 8 ml of FACS buffer was added to stop the RBC lysis reaction. Cells were spinned at 600 g for 5 minutes and the resuspended cells were passed through the cell strainer (40 µm) into 50 ml tubes and the cell concentrations were adjusted to 1-5x10^6^ cells/ml.

To make single cell suspension from lung, lung lobs were collected and kept in DMEM medium-low containing 10% fetal bovine serum (FBS) on ice. Next, lung was washed twice with PBS and then micro-dissected into 2 mm pieces and digested in 2 ml PBS containing 20 mM HEPES, 5% FBS, 60 U/ml DNAse1 (Sigma, # 11284932001), and 450 U/ml collagenase type I (Sigma, # SCR103) at 37°C for 45 minutes with shake every 15 minutes. Then the mixture was passed through a 70 µm strainer into 50 ml tube. Cells were spinned at 600 g for 7 minutes and the lung cells were resuspend in in ice-cold FACS buffer.

Cells were stained with anti-CD3, CD4, CD25, CD127, CD8, and CD45 (BD Bioscience, San Jose, CA) for 15 minutes at 4°C, and washed twice with FACS buffer before running on BD™ LSR II flow cytometer. For cytokine detection, isolated splenocytes and lung cells were incubated in RPMI-1640 complete medium (supplemented with 10% FBS, 50 μM 2-ME, 10 mM L-glutamine, 10 mM HEPES, 1 mM sodium pyruvate, 100 U/mL pen/strep, and β-​mercaptoethanol) at 37°C with cell stimulation cocktail (plus protein transport inhibitors) (#00-4975-93, Invitrogen) for 5 hours. Cells were stained with previously mentioned surface markers followed by intracellular staining with anti-IL-10, and anti-IL-17A. Data was analyzed on Flowjo v10.

### CD4^+^CD25^high^CD127^low^ Treg Purification and RNA-Seq

Splenocytes were stained with anti-CD4, CD25, and CD127 (BD Bioscience, San Jose, CA) for 15 minutes at 4°C and washed twice with FACS buffer. Cell-sorting experiments were performed using an Aria Cell Sorter (BD Biosciences) at Temple University Lewis Katz School of Medicine Flow Cytometry Core. The CD4^+^CD25^high^CD127^low^ cells were sorted directly into TRIzol, and total RNAs from four groups (n = 2) were extracted using miRNeasy Mini kit (Qiagen) following the manufacturer instruction.

RNA-Seq was performed by BGI (North America) (24). Using the fragmentation buffer, the mRNAs were fragmented into short fragments (about 200–500 nucleotides), then the first-strand cDNA was synthesized by random hexamer primer using the fragments as templates, and dTTP was substituted by dUTP during the synthesis of the second strand. Short fragments were purified and resolved with elution buffer for end repair and single nucleotide A (adenine) addition. After that, the short fragments were connected with adaptors, and then the second strand was degraded finally using uracil *N*-glycosylase (2). After agarose gel electrophoresis, the suitable fragments were selected for polymerase chain reaction (PCR) amplification as templates. During the quality control steps, an Agilent 2100 Bioanalyzer and ABI StepOnePlus Real-Time PCR System (Thermo Fisher) were used for quantification and qualification of the sample library. At last, the library was sequenced with an Illumina HiSeq4000 using the PE100 strategy. Primary sequencing data produced by the Illumina Hiseq4000, called raw reads, were filtered into clean reads by removing adaptor containing and low-quality reads by BGI in-house software. A reference annotation–based assembly method was used to reconstruct the transcripts by TopHat (v2.0.10) and Cufflinks (v2.1.1), and background noise was reduced using fragments per kilobase million and coverage threshold.

### Statistical Analysis of RNA-Seq Data

Data analysis was carried out using the statistical computing environment R, the Bioconductor suite of packages for R, and RStudio (24). Raw data were background-subtracted, variance-stabilized, and normalized by robust spline normalization. Differentially expressed genes were identified by linear modeling and Bayesian statistics using the Limma package (25). For comparisons between two groups, moderated *t* test was used for evaluation of statistical significance. All original RNA-seq data were deposited in the NCBI’s Gene Expression Omnibus database (GSE198210).

### Ingenuity Pathway Analysis

We utilized Ingenuity Pathway Analysis IPA, Qiagen, https://www.qiagenbioinformatics.com/products/ingenuity-pathway-analysis/) to characterize the clinical relevance and molecular and cellular functions related to the identified genes in our microarray analysis. Differentially expressed genes were identified and uploaded into IPA for analysis. The core and pathways analysis was used to identify molecular and cellular pathways, as we previously reported (26–28). P value < 0.05, and |Z-score|≥1 was set as cut-off in this study. Of note, pathways with |Z-score|≥2 were designated as significantly enfluenced (29).

## Results

### SM + M Treatment Decreased CD4^+^CD25^high^CD127^low^ Treg Cells in the Lung and Lymph Nodes

**Figure 1:**
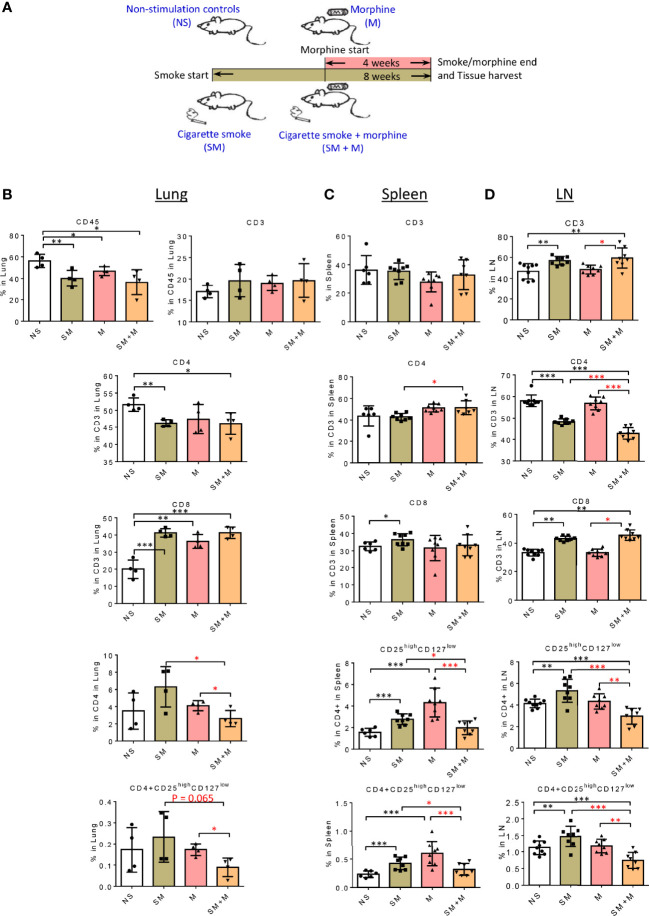
SM + M treatment decreased CD4+CD25^high^CD127^low^ Treg cells in the lung and lymph nodes. **(A)** Schematic representation of stimulations in four groups of male mice: 1) non-stimulation controls (NS); 2) smoking (SM); 3) morphine (M); and 4) smoking plus morphine (SM + M). B-D. The flow cytometry detections were performed for analyzing the populations of CD45 (lung only), CD3, CD4, CD8, CD25^high^CD127^low^ Treg in **(B)** lung (n = 4), **(C)** spleen, and **(D)** lymph node (LN) (n = 8). Bar graphs indicate the percentage of each subset in its parent population. Percentage of CD25^high^CD127^low^ Treg in all cells from lung, spleen and LN were calculated and showed in the last row. (T-test, *p < 0.05, **p < 0.01, ***p < 0.001, * in black represents the difference between SM, M or SM+M and NS control, * in red represents the difference between SM+M and SM or M).

In an effort to examine the effects of SM and M on the regulatory T cell component of the imimune system, we first profiled T cell subsets by flow cytometry in the lung in animals subjected to SM, or chronic treatment with M, or the combination as described in **Figure 1A**. The results show (**Figure 1B** and **Suplementery S1**) that SM, M and SM + M decreased numbers of CD45^+^ total leukocytes in the lung compared to the control group but did not change the proportion of T cells in this organ. Further characterization of the T cell compartment, showed that each of the treatments increased the proportion of the CD8^+^ T cell subset, with a coinciding decrease in the CD4^+^ subset in the SM and SM + M but not in M group. Next, we examined the CD25^high^CD127^low^ Treg cell frequency in both subsets and found that SM, M and SM + M did not significantly change Treg in the CD4^+^ or CD8^+^ T cell subsets in the lung compared to the control group. However, SM + M decreased CD4^+^CD25^high^CD127^low^ Treg cells compared to either M treatment alone or SM treatment.

We also examined the T cell compartment in spleen and the results show (**Figure 1C** and **Suplementary S1**) that SM treatment alone slightly but significantly increased CD8^+^ subset compared to the control. In addition, SM + M slightly but significantly increased the CD4^+^ subset compared to the SM treated group. However, no significant changes were observed in the M treated group. Furthermore, we found that SM and M treatments alone significantly increased the percentage of CD25^high^CD127^low^ Treg cells in in the spleen, in contrast to the SM + M treatment. Moreover, we examined the LN, and here again the SM and SM + M modestly, but significantly, increased the percentage of CD3^+^ T cells, but decreased the proportionof CD4^+^ T cell subset with a modest increase in the percentage of the CD8^+^ subset. In contrast to the lung, there was a significant reduction in the percentage of CD4^+^CD25^high^CD127^low^ Treg cells (**Figure 1D** and **Suplementary S1**), and both the SM alone and M alone failed to exhibit this effect. As with the lung, the exposure to SM + M caused an decrease in CD8^+^CD25^high^CD127^low^ cells in LN.

Taken together, these results have demonstrated that SM, M and SM + M did not change CD4^+^CD25^high^CD127^low^ Treg cells in lung but SM + M decreased CD4^+^CD25^high^CD127^low^ Treg cells compared to treatments with either SM or M alone. In addition, SM and M did, but SM + M did not, increase CD4^+^CD25^high^CD127^low^ Treg cells in spleen. Finally, SM increased, SM + M decreased, but M did not change, the proportion of Treg cells in LN.

### Morphine Is Stronger Than Cigarette Smoke in Re-Shaping Treg Cell Transcriptomes, and SM + M Have Synergy in Modulating Treg Cell Transcriptomes

Previuos studies reported that Treg cells are weakened in patients with COPD (10). Cigarette smokers with COPD also have significantly fewer Treg cells and a lower level of Foxp3 mRNA in the lung than healthy smokers (30). We previously reported that chronic M administration increased numbers of circulating Treg cells and functional Th17 cells (31), suggesting that even with increased Treg cell numbers, M may weaken Treg function in suppressing Th17. We hypothesized that SM, M, or SM + M may re-shape Treg cell transcriptomes. To test this hypothesis, we isolated splenic CD4^+^CD25^high^CD127^low^ Treg cells and performed RNA-Seq on the isolated cells. Of note, to preserve Treg cell RNA quality, we did not use Treg cell-specific transcription factor Foxp3 as an intracellular staining marker for the Treg. Based on the results (**Table 1**), we found that SM alone significantly modulated 648 genes with 211 genes upregulated and 437 genes downregulated. The analysis also showed that morphine alone significantly modulated 902 genes with 233 genes upregulated and 669 genes downregulated, and SM + M significantly modulated 1,630 genes with 630 genes upregulated and 1,000 genes downregulated. Moreover, SM + M significantly modulated 1507 genes with 743 genes upregulated and 764 genes downregulated compared to the M treated alone. Finally, SM + M significantly modulated 710 genes with 349 genes upregulated and 361 genes downregulated compared to SM alone.

**Table 1:** CD4+CD25^high^CD127^low^ regulatory T cells (Treg) RNA-Seq experiments led to identification of numerous differentially expressed genes (DEGs) in Treg in four groups of mice (n = 2).

Comparison of groups	Genes annotated	Genes with P.Value <0.05	Genes with |log2FC| >1	
SM vs NS	19121	1105	648	↑ 211
				↓ 437
M vs NS		1171	902	↑ 233
				↓ 669
SM+M vs NS		2539	1630	↑ 630
				↓ 1000
SM+M vs M		2196	1507	↑ 743
				↓ 764
SM+M vs SM		912	710	↑ 349
				↓ 361

To determine the various stimulation effects on Treg transcriptome, the comparisons were made in five pairs: i) SM versus NS; ii) M versus NS; iii) SM_M versus NS; iv) SM_M versus M; and v) SM_M versus SM. ↑means up-regulated; ↓means down-regulated.

These results have demonstrated that *first*, the 4 week chronic M administration is stronger than the 8 week SM exposure in re-shaping Treg cell transcriptomes, potentially due to the more focused receptor-signaling pathways of M than SM; and *second*, SM plus M have synergistic effects in modulating Treg transcriptomes.

### SM, M, and SM + M Downregulate IL-10 Expression, and SM + M Increase Th17 Transcription Factor (TF) Rorc Expression in Treg Cells

**Figure 2:**
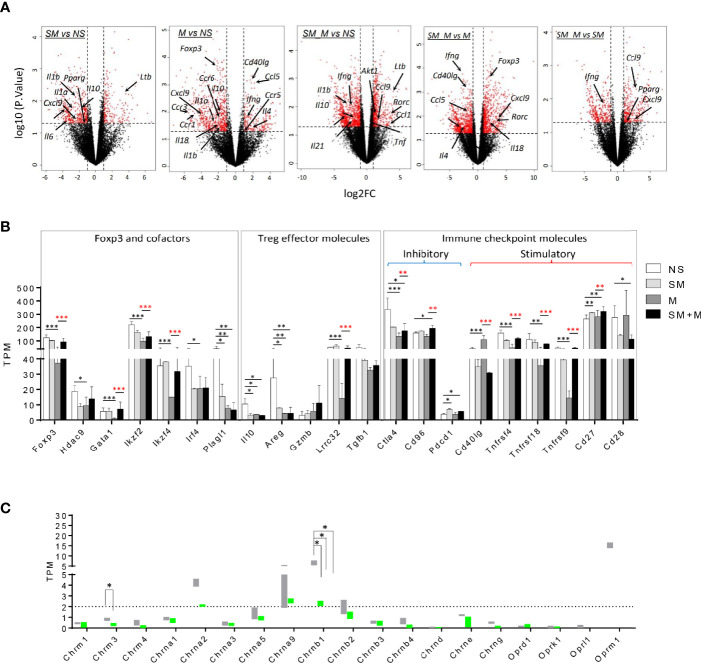
Morphine (M) is stronger than cigarette smoke (SM) in re-shaping Treg cell transcriptomes and SM + M have synergy in modulating Treg cell transcriptomes. **(A)** Volcano plots illustrate log 10 (p-value) in relation to the log 2 (fold change) for the treatments versus control (n = 2). The differentially expressed genes with -log10 (p. value) >1.3 and |log2FC| >1 are displayed in red. Cytokines, chemokines and receptors with transcriptomic changes are labeled. **(B)** Bar graph show the level of transcripts of Foxp3 and cofactors, Treg effector molecules, and immune checkpoint molecules in Treg from four groups. **(C)** Bar graph show the level of transcripts of 15 nicotinic receptors and 4 opioid receptors in Treg from four groups. TPM, Transcripts per Million mapped reads (T test, *p < 0.05, **p < 0.01, ***p < 0.001, * in black represents the difference between SM, M or SM+M and NS control, * in red represents the difference between SM+M and SM or M).

We hypothesize that pathological conditions changes Treg characteristics, result in generating distinct cytokines, interactors/signaling pathways and chemokines. To test this hypothesis, we analyzed changes in cytokine mRNA expression in Treg cells from SM, M and SM + M treated animals. The results show (**Figure 2A**) that SM, M and SM + M treatments significanly downregulated IL-10 expression in Treg cells compared to the control. In addition, Treg cells from M-treated animals increased expression of the Th1 cytokine *Ifng*, the Th2 inducing cytokine *Il4* (32), chemokine *Ccl5* and receptor *Ccr5*, but decreased *Ccr1, Ccr3*, and *Ccr6* expression. Treg cells from the SM + sM treated animals exhibited lower *Ifng*, but increased proinflammatory cytokine tumor necrosis factor (*Tnf*), *Ccl1*, *Ccl9*, and notably, increased expression of Th17 transcription factor *Rorc*.

In adition, we found that *Foxp3* and its cofactors *Hdac9*, Gata1, *Ikzf2* (encodes Helios), *Ikzf4*, *Irf4*, and *Plagl1* were specifically inhibited following treatmen with M (**Figure 2B**), which was consistent with the previous finding that chronic M administration weakened Treg function (31). Since SM, M and SM + M significantly suppressed *IL-10* production, we next investigated the levels of other Treg effector molecules, which are critical for Treg cell function (33). As expected, EGF-like growth factor amphiregulin (Areg), which confers Treg cell suppressive function *via* the EGFR/GSK-3β/Foxp3 axis, was significantly downregulated in SM, M and SM + M Treg cells. *Lrrc32* (encodes glycoprotein-A repetitions predominant, GARP), which is critical for tethering TGF-β to the cell surface, was specifically decreased with M treatment. No change was observed in the expression of Gzmb (encodes granzyme B) and Tgfb1 (encodes TGF-β1). Moreover, we examined the expression of immune checkpoint molecules, and the results show that SM + M increased two co-inhibitory checkpoints *Cd96* (93) and *Pdcd1* (PD1) and decreased both co-inhibitory *Ctla4* and co-stimulatory *CD28.* Morphine treatment alone decreased three TNF receptor superfamily members, *Tnfrsf4* (OX40), *Tnfrsf18* (GITR), *Tnfrsf9* (4-1BB), but SM + M did not. Finally, SM increased co-stimulatory checkpoint *Cd40lg* and the induction was absent with the combined administration of SM plus M.

### SM, M and SM + M Downregulate the Expression of Nicotinic Acetylcholine Receptor Chrnb1

In order to assess the expression of the nicotinic receptor and opioid receptor on Treg cells, we analyzed the expressions of three muscarinic acetylcholine receptor subunits, 12 nicotinic acetylcholine receptor (nAChR) subunits (34) and four opioid receptors (35) in SM, M, and SM + M treated Treg cells. The results show (**Figure 2C**) that among the nicotinic receptor subunits examined, nAChR *Chrna1*, *Chrna2*, *Chrnb1*, *Chrmb2* have comparably higher expression levels than other subunits, suggesting that these subunits might be the dominant structural conponents on Treg cells. Among these, Chrnb1 was significantly downregulated with SM, M and SM + M treatment. In addition, M treatment specifically decreases the expression of muscarinic acetylcholine receptor *Chrm3.* On the other hand, analysis of the opioid receptor transcripts shows that Oprm1 exhibits a higher basal level among these receptors on Treg cells, but no significant change were observed between the treatment groups.

### SM + M Have Synergistic Effects to Activate TWEAK, PI3K/AKT and OXPHOS Pathways on Treg Cells

**Figure 3:**
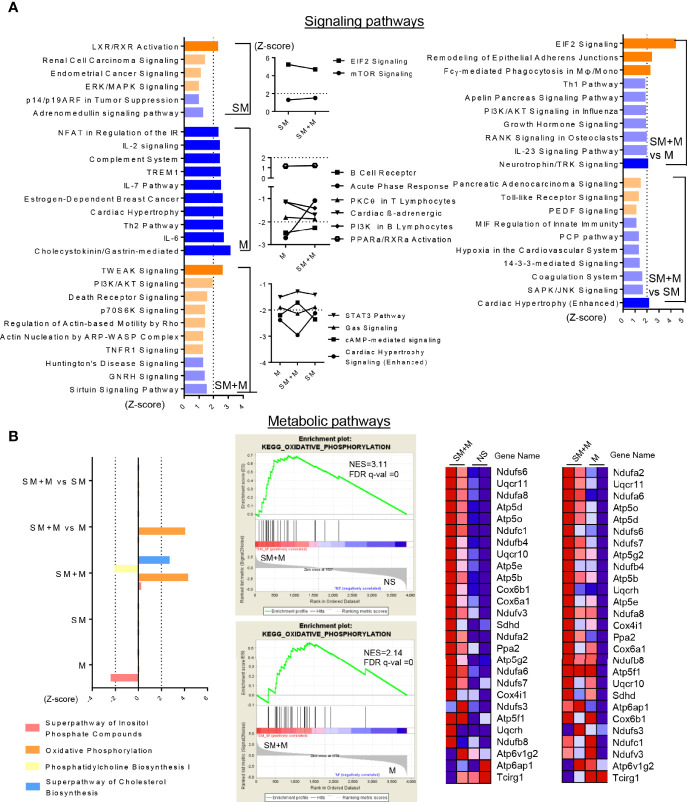
SM + M have synergistic effects to activate TWEAK, PI3K/AKT and OXPHOS pathways on Treg cells. **(A)** Ingenuity Pathway Analysis (IPA) identify the signaling pathways based on the differentially expressed genes (with P value < 0.05) from each comparisons. Bar graph (left) show the top 10 pathways (ordered by |Z-score|) only modulated upon SM, M and SM + M treatment versus control (P value < 0.05, and |Z-score|≥1 was set as cut-off in this study). Plot graph (middle) show overlapped pathways modulated in SM + M and SM or/and M. Bar graph (right) show top pathways modulated upon SM + M versus SM or M alone. Bar in dark orange (Z-score ≥2) represent activation of the pathway and in dark blue (Z-score ≤ -2) represent inhibition. **(B)** Ingenuity Pathway Analysis (left) identify the metabolic pathways modulated upon treatments. Gene set enrichment assay (GSEA, middle) also identify the expression dataset of SM + M are significantly enriched in the class of genes involved in KEGG OXPHOS pathway. Heatmaps on the right display the gene change associate with the activation of OXPHOS in SM + M when compared with NS or M alone. (NES, normalized enrichment score; FDR, false discovery rate).

In order to have a more comprehensive assessment of the modulation that SM, M and SM + M induced in Treg cells, we used IPA to determine the signaling pathways related to all of the transcriptomic reprogramming of Treg cells treated by SM, M, and SM + M. As shown in **Figure 3A**, SM + M preferentially activated TWEAK (TNF-like weak inducer of apoptosis) and phosphatidylinositol 3-kinase/protein kinase B (PI3K/AKT) (Z-score =1.964) signaling. In contrast with Treg cells from SM + M treated animals, pathways for IL-2 and IL-6 signaling, Th2 activation, and TREM-1 signaling were all inhibited in M treated Treg cells. SM treatment selectively activated LXR/RXR signaling. Moreover, several significantly modulated pathways were shared by SM + M, SM, and/or M alone. For example, SM + M and M both activated mTOR signaling. SM + M and SM both activated the PPARa/RXRa pathway, and inhibited both PKCθ signaling, and the STAT3 pathway.

Sterol metabolism has been linked to the anti-inflammatory response in CD4^+^ T cells, therefore, we examined the metabolic pathways significantly affected by SM, M, and SM + M (**Figure 3B**). Our results showed that SM + M treatment activated oxidative phosphoration (OXPHOS), and superpathway of cholesterol biosynthesis, while suppressed phosphatidylcholine biosynthesis. In addition, using gene set enrichment analysis (GSEA), we also found genes activating OXPHOS were significantly enriched in SM + M Treg when compared to control or M Treg.

### Treatment With S+M Stimulated IL-17 Production in CD4^+^CD25^high^CD127^low^ Treg Cells From Spleen and Lung

**Figure 4:**
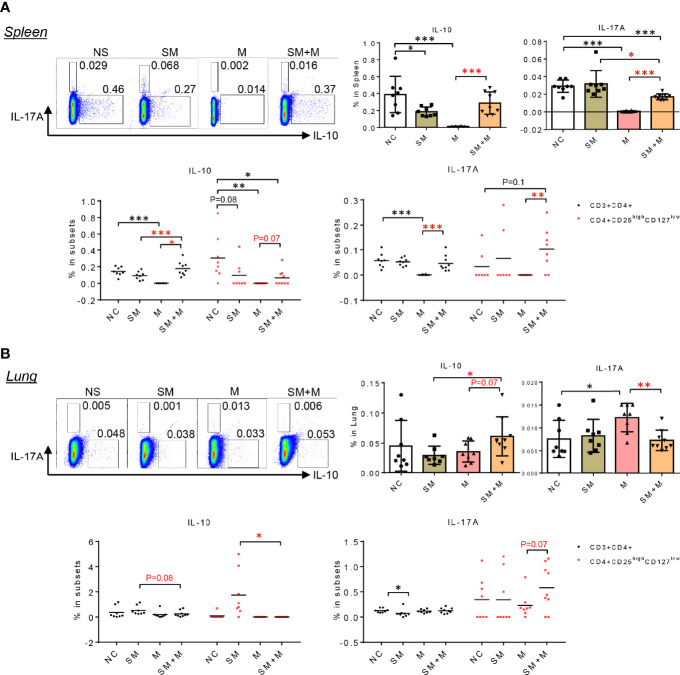
SM + M stimulated IL-17A production in CD4+CD25^high^CD127^low^ Treg cells from spleen and lung. **(A)** Representative flow cytometry gating and quantitative bar graph show the IL-17A and IL-10 production in splenocytes upon treatments. Dot plot indicate the frequency of IL-10 and IL-17A producing cells in CD3+CD4+ T cells and Treg cells. **(B)** Representative flow cytometry gating and quantitative bar graph show the IL-17A and IL-10 production in lung cells upon treatments. Dot plot indicate the frequency of IL-10 and IL-17A producing cells in CD3+CD4+ T cells and Treg cells. (n = 8) (T-test, *p < 0.05, **p < 0.01,***p < 0.001, * in black represents the difference between SM, M or SM+M and NS control, * in red represents the difference between SM+M and SM or M).

To test the hypothesis that SM, M and SM + M weakened Treg function and SM + M-Treg cells exhibited IL-17-biased plasticity, which was suggested by the RNA-seq results, we determined the numbers of cells producing IL-10 and IL-17A in splenocytes and lung cells using flow cytometry. The results show (**Figure 4A**) that splenic IL-10 production was significantly decreased in animals following SM and M treatments, but was not significantly altered by SM + M. However, in the splenic CD4+CD25^high^CD127^low^ Treg subsets, IL-10 production was inhibited not only by SM, and M, but also by SM + M treatment, which was consistent with the RNA-Seq results (**Figure 2A**). Moreover, we also verified that IL-17A production in the splenic CD4+CD25^high^CD127^low^ Treg subsets was induced in SM + M Treg cells but not in the Treg cells from SM or M treated mice. Of note, M treatment alone significantly suppressed both IL-10 and IL-17A production in the spleen and in CD3^+^CD4^+^ splenocytes.

Next, we examined IL-10 and IL-17A cytokine production in lung cells, and found that SM + M treatments significantly increased IL-10 production in lung cells compared to SM alone (**Figure 4B**). However, elevated levels of IL-10 were not observed in the Treg subsets in the lung. Furthermore, M, but not SM + M, treatment induced IL-17A production in lung cells. However, Treg cells from SM + M treated mice showed a slight but not signicant increase in IL-17 production compared to in the CD4^+^CD25^high^CD127^low^ subset from M treated animals. It is worth noting that we did not analyze cells that produce both IL-10 and IL-17A in these experiments because of the low level of double-positive events.

## Discussion

Treg cells have a great therapeutic potential for many diseases including systemic lupus erythematosus, organ-specific autoimmune diseases (36) (type I diabetes, psoriasis, myasthenia gravis, inflammatory bowel disease, and multiple sclerosis), transplantation, cancers (37, 38), and cardiovascular disease (39–41). Altered Treg cell numbers have been reported in tobacco use related diseases including increased Treg cell numbers in pulmonary lymphocyte follicles of moderate COPD patients (8), increased Foxp3^+^ Treg cells in large airways but decreased in small airways (main pathological sites in COPD) (9, 10), and decreased Treg cell numbers in the blood of patients with severe COPD (13), suggesting that the chronic inflammation in these patients may be promoted by lack of this regulatory cell population. In addition, studies revealed that M use alters T cell compartmentation and function, including the Treg cell component (20, 42). Our study observed that both SM and M alone increased the proportion of Treg cells in spleen, and SM increased the percentage of Treg cells in LN. Furthermore, we identified that SM + M decreased Treg cells in lung, spleen, and LN compared to either M alone or SM alone. The greater strength of the combination of SM and M is consistent with our previous studies suggesting that these treatments are more potent inhibitors of Treg cells than either SM or M alone (15).

In an effort to understand the molecular basis for the effects of SM and M, and the combination, we conducted RNAseq analysis of the Treg cells. Our results revealed that these treatments have a number of important influences on the transcripts, and molecular pathways that control Treg cell function. First, while we found that M is potent in re-shaping Treg transcriptomes, the combination of SM + M exhibit synergistic activity in modulating the Treg cell transcriptome. For example, we observed and verified that SM + M, as well as M and SM alone, downregulated IL-10 gene expression and protein production. Given the important role of this cytokine in the function of Treg cells (43–46), these results suggest that SM and M down-regulate both the numbers and functional activity of these important regulatory cells. In addition, we found that SM + M increased Th17 transcription factor *Rorc* and the IL-17A generation, which suggested that SM + M Treg cells exhibited IL-17-biased plasticity (47).

In addition, data from RNAseq analysis showed that M Treg cells decreased the expression of Foxp3 and its cofactors, suggesting a defective regulatory network, which may accelerate the expression of pro-inflammatory chemokine expression by T cells, monocytes and macrophages (17). In addition, M decreased the expression of co-stimulatory checkpoints *Tnfrsf4*, *Tnfrsf9*, *Tnfrsf18*, but increased *Cd40lg* in Treg. It has been reported that M inhibited antigen-presenting abilities of dendritic cells and anti-CD40 antibodies could restore the function (48). Taking together, further work is needed to assess the CD40/CD40L interaction between dendritic cells and Treg following chronic M treatment. Meanwhile, while M decreases Ctla-4 expression, SM + M Treg cells exhibited downregulation of both Ctla-4 and CD28, the co-inhibitory and co-stimulatory checkpoint couple that compete to bind to the antigen presenting cell (APC) ligands CD80/CD86. These results suggest a dysfunction in the binding properties of APC ligands CD80/CD86 in SM + M treated animals (49).

The greater strength of the combination of SM and M was also apparent in signaling and metabolic pathways. SM + M activated TWEAK signaling in Treg, which has been reported to amplify inflammation and promote IL-17 secrection (50), leading to tissue damage and potentially impeding endogenous repair mechanisms (51). Treatment with SM + M also resulted in the activation of PI3K-AKT singaling, which is reported to deliver a cell-intrinsic negative signal to restrain Treg cell suppressive activity (52). Moreover, SM + M and M both activated mTOR signaling, which, on the one hand, triggers Treg proliferation, but also weakens the functions and stability of Treg (53, 54). Besides, mTOR also directs Treg cell polarization into other functionally plastic subpopulations (55). Metabolically, SM + M activated the OXPHOS pathway. Compared to the Th subsets, Treg cells are less reliant on glycolysis and use OXPHOS for energy production (56). In general, Treg show a decrease in glycolysis and an increase in OXPHOS, fatty acid oxidation/synthesis during their phase of suppressive function (57). However, a very recent study reported that under Th17 conditions, OXPHOS fine-tunes pathogenic Th17 and Treg cell fate decision and mitochondrial respiration determines the lineage specification of pathogenic Th17 over immunosuppressive Treg cell (58).

**Figure 5:**
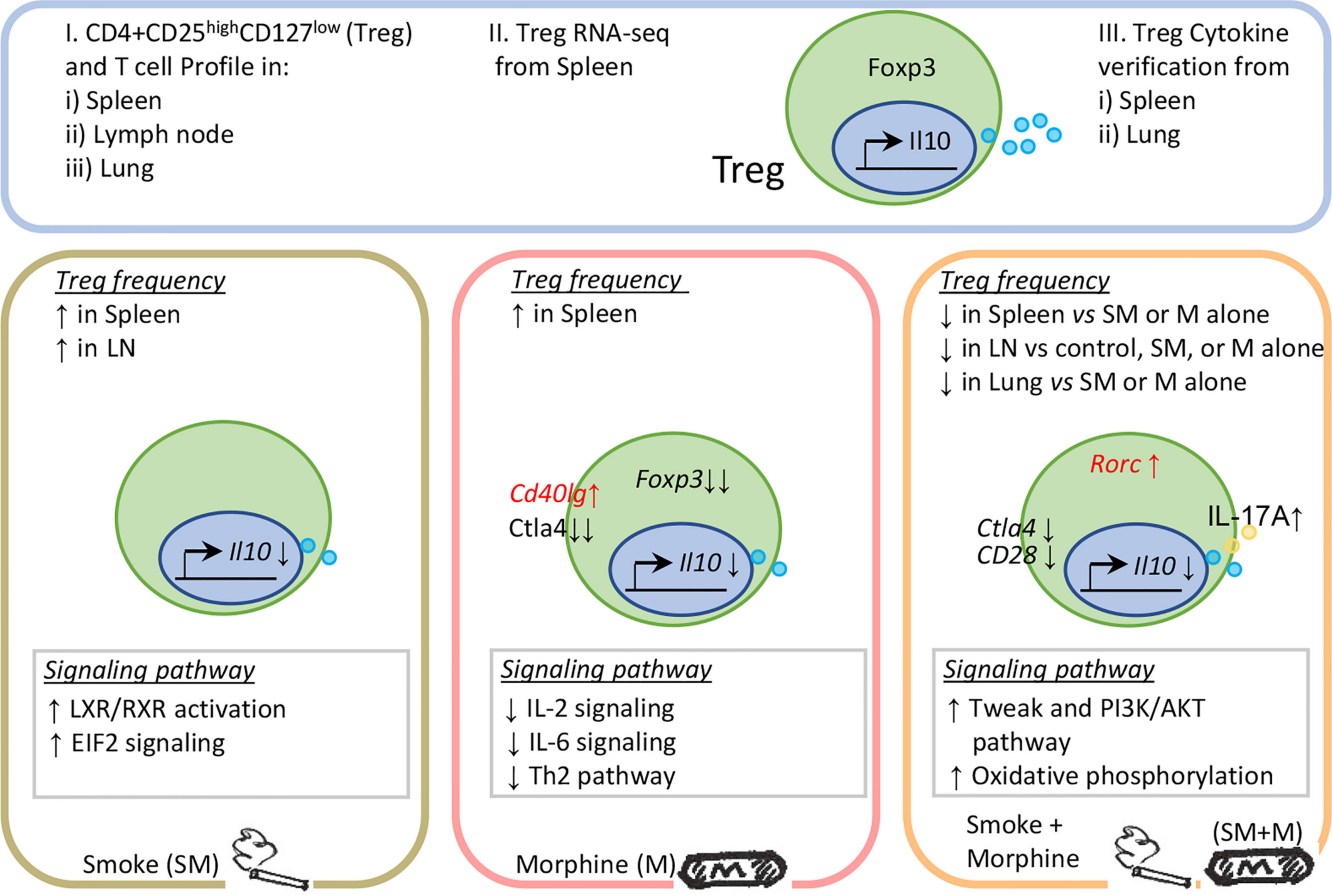
Working model: Chronic exposure to the combination of cigarette smoke and morphine decreases CD4+ regulatory T cell numbers by reprogramming the Treg cell transcriptome.

In conclusion, S + M treatment downregulated the Treg population in the lung and peripheral lymphoid organs more than cigarrette smoke or morphine, and re-programs Treg transcritptomes *via* the cell death related TWEAK pathway, PI3K-AKT/mTOR pathway and metabolic-driven OXPHOS pathway. In addition, S + M treatment induced Treg cells to exhibit Th17-biased plasticity (**Figure 5**). Our findings provide novel insights on the roles of cigarette smoke, morphine and the combination in reprogramming Treg transcriptomes as well as novel targets for the future therapeutic interventions involving immunosuppression, including human immunodeficiency virus (HIV) pathologies, drug abuse pathologies, COPD, cardiovascular diseases, autoimmune diseases, transplantation, cancers and tissue repair.

## Data Availability Statement

The datasets presented in this study can be found in online repositories. The names of the repository/repositories and accession number(s) can be found below: NCBI, accession ID: GSE198210.

## Ethics Statement

The animal study was reviewed and approved by Animal Care and Use Committee (IACUC) of Temple University Lewis Katz School of Medicine.

## Author Contributions

YS, WC and AK performed animal works and YS carried out flow cytometry and RNAseq data gathering, data analysis and prepared tables and figures. WC, KX, AK, FS, YL, XJ, GC, HW aided with analysis of the data. XY and TR supervised the experimental design and manuscript writing. All authors read and approved the final manuscript.

## Funding

Our research activities are supported by grants from the National Institutes of Health (NIH) (HL131460, HL132399, HL138749, HL147565, P30 DA13429, RO1 DA040619, RO1 DA049745). The content in this article is solely the responsibility of the authors and does not necessarily represent the official views of the NIH.

## Conflict of Interest

The authors declare that the research was conducted in the absence of any commercial or financial relationships that could be construed as a potential conflict of interest.

## Publisher’s Note

All claims expressed in this article are solely those of the authors and do not necessarily represent those of their affiliated organizations, or those of the publisher, the editors and the reviewers. Any product that may be evaluated in this article, or claim that may be made by its manufacturer, is not guaranteed or endorsed by the publisher.
