# Supplementary figures and images for: Chronic Exposure to the Combination of Cigarette Smoke and Morphine Decreases CD4+ Regulatory T Cell Numbers by Reprogramming the Treg Cell Transcriptome
Source: Front Immunol. 2022 Apr 20;13:887681. doi: 10.3389/fimmu.2022.887681 (PMC9065607; doi:10.3389/fimmu.2022.887681)

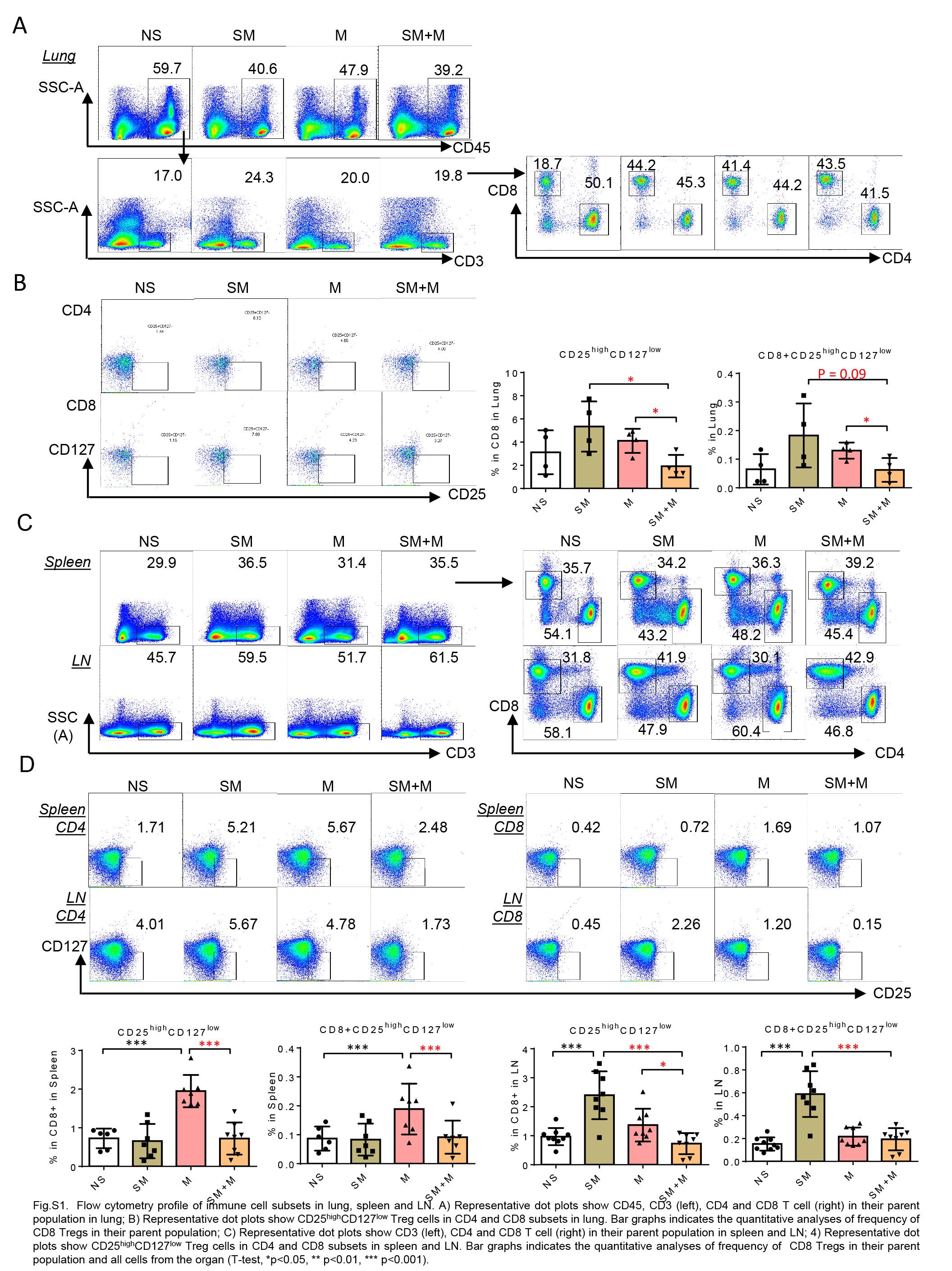

Supplement: Supplementary file 1 [file Image_1.tif]
